# International comparisons of the management of patients with non-ST segment elevation acute myocardial infarction in the United Kingdom, Sweden, and the United States: The MINAP/NICOR, SWEDEHEART/RIKS-HIA, and ACTION Registry-GWTG/NCDR registries^☆^^☆☆^

**DOI:** 10.1016/j.ijcard.2014.04.270

**Published:** 2014-08-01

**Authors:** R.L. McNamara, S.C. Chung, T. Jernberg, D. Holmes, M. Roe, A. Timmis, S. James, J. Deanfield, G.C. Fonarow, E.D. Peterson, A. Jeppsson, H. Hemingway

**Affiliations:** a Yale University School of Medicine, Cardiovascular Medicine, New Haven, CT, USA; b Farr Institute of Health Informatics Research @ UCL Partners, University College London, London, UK; c Dept of Medicine (Huddinge), Cardiology, Karolinska Institutet, and Dept of Cardiology, Karolinska University Hospital, Stockholm, Sweden; d Duke Clinical Research Institution, Duke University Medical Center, Durham, NC, USA; e National Institute for Health Research, Biomedical Research Unit, Barts Health London, UK; f Dept. of Medical Sciences, Cardiology and Uppsala Clinical Research Center, Uppsala University, Uppsala, Sweden; g University College London, London, UK; h Ronald Reagan-UCLA Medical Center, Los Angeles, CA, USA; i Sahlgrenska University Hospital, Sahlgrenska Academy, University of Gothenburg, Gothenburg, Sweden

**Keywords:** ACEI, angiotensin converting enzyme inhibitors, ACS, acute coronary syndrome, ACTION Registry-GWTG, The Acute Coronary Treatment and Intervention Outcomes Network Registry — Get With The Guidelines, ARB, angiotensin receptor blockers, CABG, coronary artery bypass grafting, ECG, electrocardiogram, GRACE, Global Registry of Acute Coronary Events, MI, myocardial infarction, MINAP, Myocardial Ischemia National Audit Project, NCDR, National Cardiovascular Data Registry, NSTEMI, non-ST segment elevation myocardial infarction, NICOR, National Institute for Cardiovascular Outcomes Research, PCI, percutaneous coronary intervention, RIKS-HIA, Register of Information and Knowledge About Swedish Heart Intensive Care Admissions, SWEDEHEART, Swedish Web-system for Enhancement and Development of Evidence-based care in Heart disease Evaluated According to Recommended Therapies, UK, England/Wales, US, United States, Acute myocardial infarction, International comparisons, Clinical registries, Treatment

## Abstract

**Objectives:**

To compare management of patients with acute non-ST segment elevation myocardial infarction (NSTEMI) in three developed countries with national ongoing registries.

**Background:**

Results from clinical trials suggest significant variation in care across the world. However, international comparisons in “real world” registries are limited.

**Methods:**

We compared the use of in-hospital procedures and discharge medications for patients admitted with NSTEMI from 2007 to 2010 using the unselective MINAP/NICOR [England and Wales (UK); n = 137,009], the unselective SWEDEHEART/RIKS-HIA (Sweden; n = 45,069), and the selective ACTION Registry-GWTG/NCDR [United States (US); n = 147,438] clinical registries.

**Results:**

Patients enrolled among the three registries were generally similar except those in the US who were younger but had higher rates of smoking, diabetes, hypertension, prior heart failure, and prior MI than in Sweden or in UK. Angiography and percutaneous coronary intervention (PCI) were performed more often in the US (76% and 44%) and Sweden (65% and 42%) relative to the UK (32% and 22%). Discharge betablockers were also prescribed more often in the US (89%) and Sweden (89%) than in the UK (76%). In contrast, discharge statins, angiotensin converting enzyme inhibitors/angiotensin receptor blockers (ACEI/ARB), and dual antiplatelet agents (among those not receiving PCI) were higher in the UK (92%, 79%, and 71%) than in the US (85%, 65%, 41%) and Sweden (81%, 69%, and 49%).

**Conclusions:**

The care for patients with NSTEMI differed substantially among the three countries. These differences in care among countries provide an opportunity for future comparative effectiveness research as well as identify opportunities for global quality improvement.

## 1 Introduction

International comparisons of care of community-based populations provide valuable opportunities for identifying areas for improvement in patient care. Many aspects of the management of non-ST segment elevation myocardial infarction (NSTEMI) have a strong evidence base. Clinical trials have shown improved outcomes for an early invasive approach [1–3]; and antiplatelet therapy, betablockers, statins, and angiotensin converting enzyme inhibitors (ACEI) or angiotensin receptor blockers (ARB) upon discharge are guideline recommendations [4–6] and evaluated by performance measures [7]. Prior studies have demonstrated that the use of these therapies in routine clinical practice in the United States (US) is suboptimal [8,9], especially in some subgroups [10–12], and with high variability among hospitals [13,14].

Yet, there have been few international comparisons of care for patients with NSTEMI. While populations from select sites within clinical trials [15–17] or the Global Registry of Acute Coronary Events (GRACE) [18] have been studied across various countries, these are not representative of patients seen or care received in routine community practice [19]. Larger registries with more patients per country are needed to make valid comparison among individual countries as well as enable the assessment of trends and detailed subgroup analysis. In addition, more recent data are needed to reflect the rapid change in clinical management of NSTEMI patients.

We sought to compare patterns of in-hospital treatment and use of interventional diagnostic and therapeutic procedures among patients admitted with NSTEMI from 2007 to 2010 across three national clinical registries. The Myocardial Ischemia National Audit Project (MINAP)/National Institute for Cardiovascular Outcomes Research (NICOR) [20] and the Swedish Web-system for Enhancement and Development of Evidence-based care in Heart disease Evaluated According to Recommended Therapies (SWEDEHEART)/Register of Information and Knowledge About Swedish Heart Intensive Care Admissions (RIKS-HIA) [21] attempt to collect information on all patients with acute coronary syndrome (ACS), including NSTEMI, in all hospitals providing ACS care in England/Wales (UK) and Sweden, respectively. The Acute Coronary Treatment and Intervention Outcomes Network Registry — Get With The Guidelines (ACTION Registry-GWTG)/National Cardiovascular Data Registry (NCDR) includes patients with ACS in a large but self-selected group of hospitals in the US [22].

## 2 Methods

### 2.1 Study population

The study population was drawn from all hospitals providing acute myocardial infarction (MI) care in England and Wales (236 hospitals, 137,009 patients) and in Sweden (74 hospitals, 45,069 patients) and a voluntary subset of hospitals, most with capability to perform PCI, in the United States (500 hospitals, 147,438 patients). Patients were eligible for our study if they were admitted between 1 January 2007 and 31 December 2010, and aged at least 30 years. For patients identified to have multiple admissions we used the earliest record. NSTEMI diagnosis was based on guidelines from European Society of Cardiology/American College of Cardiology/American Heart Association. Specifically, elevated troponin levels were required. The study complies with the Declaration of Helsinki and was approved by the MINAP Academic Group, the Steering group of SWEDEHEART, and research and publications committee in ACTION.

### 2.2 Patient characteristics and hospital treatment

Baseline variables of study interest include: demographic factors (age, gender), risk factors (smoking, history of diabetes and hypertension), previous heart disease (heart failure and MI), and medication and procedure use prior to hospital admission (antiplatelet, betablocker, ACEI/ARB, or statin therapy and PCI, and prior coronary artery bypass grafting (CABG)). Key hospital presentation variables included systolic blood pressure and heart rate on arrival, first hemoglobin and creatinine levels, and troponin levels; hospital procedure variables included angiography and PCI, and discharge medication variables included antiplatelet therapy (aspirin, clopidogrel/prasugrel or both), betablockers, ACEI/ARB, and statins. Regular chart review of randomly selected patients within each hospital in SWEDEHEART/RIKS-HIA demonstrated a 96.1% agreement [21]. Re-entry of data items of randomly selected patients in each hospital in MINAP/NICOR demonstrated a median agreement of 89.5% [20]. Audit of records among randomly selected hospitals in ACTION demonstrated an accuracy of 89.7% [23].

### 2.3 Statistical methods

Numerical data are summarized as median and interquartile range (IQR) and categorical data as frequency and percentage. The distribution of case-mix (demographics, past history, and presentation characteristics) and treatment variables was compared in the UK, Sweden and the US. To investigate the secular and age difference in acute management of patients after NSTEMI, the analyses were stratified by admission year and age groups (< 60, 60–79, ≥ 80 years old). Recognizing the difference between nationwide and voluntary registries, we conducted a sensitivity analysis, comparing the results of NSTEMI patients attending PCI hospitals only. We defined PCI hospitals as those hospitals that performed a minimum of 24 PCI in the calendar year. Analyses were performed using SAS version 9.0 or 9.2 (SAS Institute, Cary, North Carolina, U.S.A.) and IBM SPSS statistics version 20.0 (IBM, Armonk, New York, U.S.A.).

## 3 Results

### 3.1 Patient demographic and clinical characteristics

The overall demographic and clinical characteristics for the patients in the three registries showed many similarities, but some notable differences were present (Table 1). The patients were youngest in the US yet these individuals had higher rates of smoking, diabetes, hypertension, prior heart failure, and prior MI than those in Sweden or in UK.

Patients in Sweden presented to the hospital with a somewhat higher blood pressure but lower creatinine level (Table 2). In the UK and Sweden, troponin levels, which likely represent a mix of initial and peak levels, were similar. Initial troponin levels were lower in the US than the mix of initial and peak levels in the other two countries, but peak levels were higher. Overall, electrocardiogram (ECG) abnormalities were comparable across the registries; of note, the UK found more patients with T wave abnormalities and the US more patients with non-ST segment abnormalities.

### 3.2 Hospital treatment

Overall, angiography was performed more often in the US (76%) and Sweden (65%) than in the UK (32%) (Table 3). Similarly, PCI was performed more in the US (44%) and Sweden (42%) than in the UK (22%).

Prescription of dual antiplatelet therapy was similar across the countries in patients who received a PCI (Table 4). However, patients who did not receive a PCI were discharged on dual antiplatelet therapy more often in the UK (71%) than in Sweden (49%) and in the US (41%) (Table 4). UK physicians prescribed betablockers at discharge least often but statins and ACEI/ARB most often; regardless of whether a patient received a PCI (Tables 3 and 4).

### 3.3 Time trends

**Fig. 1:**
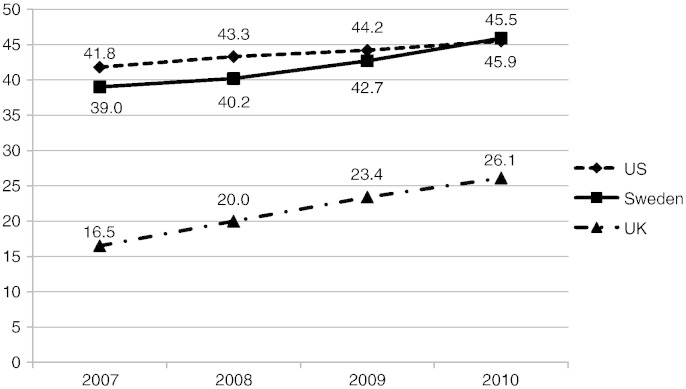
In-hospital percutaneous coronary intervention (2007–2010). % — the percentage of patients with NSTEMI who underwent percutaneous coronary intervention during the hospitalization for each of the four years of the study for each registry.

From 2007 to 2010, angiography and PCI increased in each country (Table 3 and Fig. 1), most substantially in the UK. Betablocker, dual antiplatelet, and ACEI/ARB use increased mildly in the UK. Prescription of dual antiplatelet agents, statins, and ACE/ARB increased in Sweden. The time trends showed a relatively stable use for each medication in the US, except a slight increase in use of statins.

### 3.4 Age groups

**Fig. 2:**
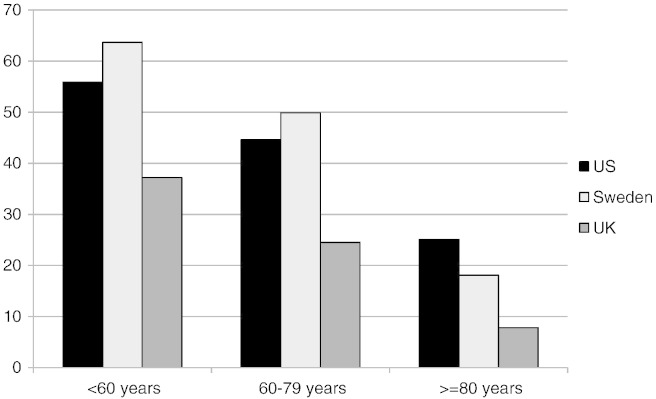
Percutaneous coronary interventions in in-hospital, by age (2007–2010). % — the percentage of patients with NSTEMI who underwent percutaneous coronary intervention during the hospitalization over the four years of the study for each registry by age group, 60 years, 60–79 years, and ≥ 80 years.

When stratified into three age groups – < 60 years, 60–79 years, and ≥ 80 years – the differences in PCI use were most marked in Sweden (Fig. 2), with younger patients more often receiving PCI than older patients. Notably, PCI in patients < 60 years and in 60–79 years was higher in Sweden than in the US; however, PCI in patients ≥ 80 years was lower in Sweden than in the US. Patterns of medication use in the age subgroups were not consistent across medications (Appendix A).

## 4 Discussion

In this comparison of ongoing national registries from the UK, Sweden, and the US, we found that in-hospital management in terms of interventional procedures for NSTEMI patients was more aggressive in the US and Sweden than in the UK. However, this gap appears to be narrowing over time. In addition, we found that, with the exception of betablockers, secondary prevention medications were more commonly prescribed in the UK at discharge than in Sweden or in the US. In particular, dual antiplatelet use in patients who did not undergo an in-hospital PCI was highest in the UK.

### 4.1 Validity of comparing national registries: case-mix

Understanding the case-mix of patients who are enrolled within each of these national registries is critical for valid international comparisons of management. Overall, the baseline characteristics of the patients in the three registries were reasonably similar. The younger age of the patients in the US may reflect the type of hospitals that participate in the registry. The higher prevalence of smoking, hypertension, previous heart failure and previous MI in the US may indicate different patient populations or may indicate a greater tendency to diagnose conditions in the US. The increased prior use of medications and procedures in the US and Sweden compared with the UK is deserving of further study. The presentation characteristics (heart rate, systolic blood pressure, hemoglobin, creatinine, troponin, and ECG abnormalities) from each country suggest a reasonably similar infarction severity.

### 4.2 Validity of comparing national registries

Importantly, we found that PCI use in all hospitals in Sweden by 2010 matched that in the self-selected subset of hospitals in the US registry. Management may be different in those submitting to ACTION hospitals than in those who do not. A comparison using only data from PCI-capable hospitals in each country, eliminating some of the hospital-level selection bias, showed similar results (Appendix B). In addition, the finding of similarly high PCI rates in an unselected registry (Sweden) and a selected registry (US) makes the differences seen in other aspects of care more informative; it acts as a ‘positive control’. (If all aspects of care differed in each country, selection bias would be more plausible.) One explanation for the comparably high rates of PCI in Sweden is the increased emphasis on system-wide quality improvement in that country [24,25]. Interestingly, the use of angiography was higher in the US than in Sweden, but PCI rates were remarkably similar. This similarity suggests that the use of PCI is not simply a function of fee-for-service model in the US because the UK and Sweden have few financial incentives for clinicians. Further studies are needed to better explain these practice patterns.

### 4.3 Procedures: trends, age

Between 2007 and 2010 the UK demonstrated a steeper increase in in-hospital procedures consistent with concerted efforts to increase PCI capability in the country [26]. Whereas the overall use of PCI was similar in the US and Sweden, the use in age groups differed considerably. Sweden showed the greatest impact of age on PCI use, with over 60% of those less than 60 years receiving PCI, while less than 20% of those greater than or equal to 80 years (Fig. 2). In contrast, in the US, those less than 60 years received PCI only 56% of the time while those less than or equal to 80 years 25%. One explanation for the high use in younger patients is that the concerted quality improvement efforts in Sweden were aimed at those less than 80 years [27]. In addition, the financial incentive for PCI placement in the US, regardless of age, likely has an impact.

### 4.4 Medications

We found markedly lower use of betablockers in the UK, both prior to hospital admission and at discharge. The higher use of statins on discharge in the UK, despite a lower use of betablockers, demonstrates a selective practice pattern rather than an overall lower medication use. The explanation of this lower overall propensity for UK physicians to prescribe betablockers is not clear but would appear to be unlikely due to economic factors (betablockers being among the cheapest secondary prevention medications) and unlikely to be due to lags in diffusion of evidence implementation (betablockers being recommended in such patients for longer than other secondary prevention medications). The considerably lower use of ACEI/ARB on discharge, despite higher frequency on admission, in Sweden and the US is interesting and deserves further study. The relatively low use of dual antiplatelet therapy in those patients who did not undergo PCI in both Sweden and the US may identify an area for quality improvement.

Clinical trials have previously shown wide variation of practice among countries or groups of countries in the management of patients with myocardial infarction [15–17]. However, management patterns within a clinical trial do not necessarily reflect management in routine clinical practice [28]. Clinical registries address some of the selection bias and are valuable resources for comparison and for assessing trends in treatment [29–32]. In particular, for over six thousand patients from fourteen countries in 1999–2001, the Global Registry of Acute Coronary Events (GRACE) found marked geographic differences for interventional therapy after NSTEMI but only modest differences for oral pharmaceutical secondary prevention therapies comparing groups of countries in Europe, North and South America, and Australia [18]. Relevant to our data, for July through December 2001, the use of PCI for patients presenting with NSTEMI was 39.5% in the US, while 34.6% for those in Europe. (It was 33.5% for those in Brazil/Argentina and 25.0% for those in Australia.) However, the GRACE registry is not ongoing, did not include a nationwide cohort of sites, nor attempt to recruit consecutive patients in all sites. The current study provides larger cohorts of patients, including nearly all NSTEMIs in Sweden and the UK. In addition, the current study reflects more recent practice patterns. Of note, we compared the use of procedures and medications in all patients, regardless of guideline indication or contraindication. For instance, we provide proportion of ACEI/ARB prescription on discharge for all patients, not just those with left ventricular dysfunction. We aimed to highlight any practice variation, rather than assess quality of care. Thus, the proportion of patients receiving medication in our study is lower than reported use in eligible patients [8].

### 4.5 Limitations

While the data we present are the best available in the three countries, they have important limitations. First, the US registry is voluntary and consists of only a subset of hospitals in the US; the registries from the UK and Sweden are mandated and represent all hospitals that admit patients with AMI. Second, the processes by which NSTEMI patients within hospitals are captured into the registries may vary between countries and are not well understood. In the UK, for example, MINAP has been shown to miss some cases, and these missed cases have a higher mortality than those included in the registry [33]. All three registries are designed to include all patients admitted to the hospital. However, for each of the registries, the number of patients with acute MI missed is difficult to estimate and may differ across countries. Third, patient historical elements such as history of cerebrovascular disease and presentation elements such as left ventricular ejection fraction would also be interesting but were not collected and recorded in a standard fashion across all three registries. These data are dependent on the quality of medical record documentation and abstraction, which may vary by registry. Finally, the comparisons are not adjusted for patient case-mix. How the differences in patient characteristics influenced management needs to be addressed in future efforts.

### 4.6 Conclusion

Differences exist in the acute management of patients after NSTEMI among the patients in national registries from the UK, Sweden, and the US. Specifically, the use of invasive procedures such as angiography and PCI and of betablockers on discharge was higher in the US and Sweden than in the UK. Interestingly, these differences are decreasing over time. Conversely, the use of some medications, such as dual antiplatelet therapy in patients who did not undergo PCI, was lower in the US and Sweden. The impact of age on use of procedures and discharge medications appears highest in Sweden. Understanding the differences in patient characteristics and hospital management is critical preludes to comparing outcomes and identifying areas for improvement in each country.

## Figures and Tables

**Appendix Table A1:** Treatment over time among NSTEMI patients age < 60 years (N = 27,901 in the UK; N = 47,467 in the US), by country, N (%).

Admission year		2007	2008	2009	2010	2007–10
Received in-hospital angiography	MINAP (UK)	37.9	44.8	54.3	57.3	49.3
	RIKS-HIA (Sweden)	88.5	91.7	93.7	94.7	92.0
	ACTION (US)	86.2	88.9	90.0	91.0	89.3
Received in-hospital PCI	MINAP (UK)	30.1	34.9	39.8	42.1	37.2
	RIKS-HIA (Sweden)	61.6	63.0	64.1	66.2	63.7
	ACTION (US)	53.8	55.8	56.1	57.1	55.9
Received dual antiplatelet at discharge	MINAP (UK)	87.1	85.9	86.1	88.3	86.9
	RIKS-HIA (Sweden)	80.5	81.4	84.8	84.3	82.7
	ACTION (US)	73.7	73.6	75.2	75.6	74.7
Received single antiplatelet at discharge	MINAP (UK)	10.1	11.2	10.9	9.6	10.4
	RIKS-HIA (Sweden)	17.1	16.2	13.5	13.6	15.2
	ACTION (US)	23.1	23.7	23.8	34.2	27.1
Received beta blocker at discharge	MINAP (UK)	85.3	86.0	85.1	87.4	86.0
	RIKS-HIA (Sweden)	90.7	91.0	90.4	90.4	90.6
	ACTION (US)	90.7	90.6	90.0	90.0	90.8
Received ACEI or ARB at discharge	MINAP (UK)	83.7	84.5	85.6	87,2	85.4
	RIKS-HIA (Sweden)	63.7	65.4	66.9	71.2	66.7
	ACTION (US)	66.5	65.3	66.1	65.8	65.9
Received statin at discharge	MINAP (UK)	95.9	96.0	95.6	96.0	95.9
	RIKS-HIA (Sweden)	92.7	93.6	94.1	94.5	93.7
	ACTION (US)	87.2	88.1	89.8	89.9	89.0

**Appendix Table A2:** Treatment over time among NSTEMI patients age 60–79 years (N = 65,621 in the UK; N = 66,968 in the US), by country, N (%).

Admission year		2007	2008	2009	2010	2007–10
Received in-hospital angiography	MINAP (UK)	26.3	32.7	40.5	44.3	36.5
	RIKS-HIA (Sweden)	74.0	78.0	81.9	83.1	79.1
	ACTION (US)	77.4	80.1	82.2	82.8	81.0
Received in-hospital PCI	MINAP (UK)	18.7	23.0	25.9	29.1	24.5
	RIKS-HIA (Sweden)	46.1	48.6	51.2	53.8	49.9
	ACTION (US)	42.8	43.5	45.2	45.9	44.6
Received dual antiplatelet at discharge	MINAP (UK)	76.6	76.6	78.7	81.1	78.4
	RIKS-HIA (Sweden)	66.6	70.7	73.7	75.3	71.5
	ACTION (US)	65.0	65.1	66.9	68.3	66.7
Received single antiplatelet at discharge	MINAP (UK)	17.9	17.9	16.6	15.1	16.8
	RIKS-HIA (Sweden)	26.5	23.6	21.5	19.4	22.9
	ACTION (US)	30.7	30.5	29.4	28.0	29.4
Received beta blocker at discharge	MINAP (UK)	74.1	76.5	77.8	79.6	77.2
	RIKS-HIA (Sweden)	88.4	89.0	88.9	89.3	88.9
	ACTION (US)	91.0	89.9	89.5	89.7	90.0
Received ACEI or ARB at discharge	MINAP (UK)	80.3	80.9	82.0	83.9	81.9
	RIKS-HIA (Sweden)	68.5	71.7	74.7	76.3	72.7
	ACTION (US)	67.5	66.5	66.2	65.6	66.3
Received statin at discharge	MINAP (UK)	93.4	93.4	93.6	94.1	93.7
	RIKS-HIA (Sweden)	87.0	89.1	90.1	90.3	89.1
	ACTION (US)	83.7	85.4	87.0	87.3	86.1

**Appendix Table A3:** Treatment over time among NSTEMI patients age ≥ 80 years (N = 43,487 in the UK; N = 33,003 in the UK), by country, N (%).

Admission year		2007	2008	2009	2010	2007–10
Received in-hospital angiography	MINAP (UK)	8.1	10.3	15.3	17.7	13.1
	RIKS-HIA (Sweden)	23.3	26.2	29.4	34.2	28.1
	ACTION (US)	43.6	46.2	36.2	49.4	46.7
Received in-hospital PCI	MINAP (UK)	5.0	6.4	8.6	10.7	7.8
	RIKS-HIA (Sweden)	15.4	16.9	18.5	22.0	18.1
	ACTION (US)	23.1	24.9	25.5	27.3	25.4
Received dual antiplatelet at discharge	MINAP (UK)	64.3	64.6	66.2	68.3	65.9
	RIKS-HIA (Sweden)	41.7	48.8	53.6	55.4	49.6
	ACTION (US)	51.1	51.9	54.3	55.5	53.5
Received single antiplatelet at discharge	MINAP (UK)	27.7	26.8	26.0	24.4	26.1
	RIKS-HIA (Sweden)	43.1	37.6	34.3	32.8	37.1
	ACTION (US)	40.1	38.8	37.0	36.2	37.8
Received beta blocker at discharge	MINAP (UK)	63.7	65.6	68.9	71.4	67.6
	RIKS-HIA (Sweden)	81.3	81.4	81.0	81.6	81.3
	ACTION (US)	87.5	86.7	85.6	84.6	85.9
Received ACEI or ARB at discharge	MINAP (UK)	68.1	68.1	70.0	70.9	69.3
	RIKS-HIA (Sweden)	55.5	59.1	58.7	60.1	58.2
	ACTION (US)	62.2	59.5	57.4	57.0	58.8
Received statin at discharge	MINAP (UK)	84.8	84.1	85.3	86.2	85.1
	RIKS-HIA (Sweden)	53.4	57.4	59.2	59.95	57.4
	ACTION (US)	71.3	73.7	75.5	76.6	74.6

**Table:** 

Admission year		2007	2008	2009	2010	2007–10
Received in-hospital angiography	MINAP (UK)	32.7	37.2	44.5	48.1	41.5
	RIKS-HIA (Sweden)	65.5	67.3	69.0	70.7	68.1
	ACTION (US)	79.5	78.5	79.4	80.3	79.6
Received in-hospital PCI	MINAP (UK)	24.8	27.6	30.5	33,5	29.6
	RIKS-HIA (Sweden)	44.0	44.3	44.8	47.4	45.1
	ACTION (US)	47.9	46.2	46.3	50.0	46.8
Received dual antiplatelet at discharge	MINAP (UK)	76.1	74.7	77.9	80.7	77.6
	RIKS-HIA (Sweden)	64.9	68.0	70.8	72.0	68.9
	ACTION (US)	66.4	65.8	67.4	68.4	67.2
Received single antiplatelet at discharge	MINAP (UK)	18.9	19.5	17.1	15.4	17.5
	RIKS-HIA (Sweden)	29.7	27.4	24.5	23.0	26.1
	ACTION (US)	29.4	29.6	28.4	27.4	28.5
Received beta blocker at discharge	MINAP (UK)	74.5	75.3	77.	80.0	77.2
	RIKS-HIA (Sweden)	89.5	90.0	88.9	89.5	89.5
	ACTION (US)	90.6	89.6	89.3	89.1	89.6
Received ACEI or ARB at discharge	MINAP (UK)	77.4	77.3	79.3	81.5	79.1
	RIKS-HIA (Sweden)	67.3	69.5	70.9	73.2	70.2
	ACTION (US)	66.2	64.8	64.5	64.0	64.7
Received statin at discharge	MINAP (UK)	92.1	91.3	92.0	93.0	92.2
	RIKS-HIA (Sweden)	81.5	83.2	83.6	83.6	83.0
	ACTION (US)	83.2	84.2	85.8	86.1	85.1

**Table 1:** Baseline characteristics.

	Total NSTEMI⁎		
	UK†	Sweden	US‡
Number of patients	137,009	45,069	147,438
Number of participating hospitals	236	74	500

Demographics			
Age, years, median (interquartile range)	73 (62–82)	73 (64–82)	67 (56–78)
Female sex, %	37.5	37.6	38.7

Risk factors			
Current smoker, %	23	19.6	29.6
Diabetes, %	21.7	25.0	35.4
Hypertension, %	53.1	50.6	76.3

History of cardiovascular disease			
Heart failure, %	7.0	11.5	16.9
Myocardial infarction, %	22.8	27.8	28.8

Treatment prior to hospital admission			
Single antiplatelet, %	30.4	42.2	39.4
Dual antiplatelet, %	4.3	4.4	13.4
Betablocker, %	28.8	44.4	44.2
ACEI§ or ARB║, %	38.3	37.3	42.5
Statin, %	44.8	32.3	43.5
PCI¶, %	7.5	12.3	25.0
CABG#, %	7.2	10.4	19.0

⁎ NSTEMI = non-ST segment elevation myocardial infarction.

† UK = United Kingdom (England and Wales).

‡ US = United States.

§ ACEI = Angiotensin converting enzyme inhibitor.

║ ARB = Angiotensin receptor blocker.

¶ PCI = Percutaneous coronary intervention

# CABG = Coronary artery bypass grafting.

**Table 2:** Presentation characteristics.

		Total NSTEMI		
		UK	Sweden	US
Number of patients		137 009	45 069	147 438
Systolic blood pressure, mm Hg⁎,†		140 (121–160)	149 (130–168)	145 (125–166)
Heart rate, beat per minute⁎		80 (68–96)	79 (66–94)	83 (70–99)
Hemoglobin, g/dl⁎,‡		13.5 (12.0–14.8)	13.7 (12.5–14.8)	13.7 (12.2–14.9)
Creatinine, mmol/l⁎		95 (79–119)	86 (72–107)	97.2 (79.6–123.8)
Troponin⁎,§	T, ng/ml║	0.33 (0.13–0.91)	0.39 (0.14–1.09)	0.1 (0.03–0.4)
	I, ng/ml	1.9 (0.39–7.59)	2.0 (0.48–7.2)	0.4 (0.1–1.8)
Initial ECG or ECG determining treatment, %	Transient ST segment elevation	2.7	4.0	2.7
	ST segment depression	30.3	34.3	23.8
	Other T wave abnormalities¶	29.8	14.9	14.1
	No ST segment abnormalities#	37.2	46.8	59.4

Abbreviations same as in Table 1 with the following additions:

⁎ Median (interquartile range) for all continuous variables.

† mmHg = millimeters of mercury.

‡ g/dl = grams per deciliter.

§ Troponin values in the UK and Sweden are a mix of initial and peak. Peak troponin values in the US were 0.6 (0.2, 1.7) and 4.7 (1.5, 14.3) for troponin T and I, respectively.

║ ng/ml = nanograms per milliliter.

¶ Includes “T wave inversion” in RIKS-HIA and ACTION and “T wave changes only” in MINAP.

# Includes “Normal”, “None”, and “Other” categories.

**Table 3:** Treatment over time among NSTEMI patients, in all patients, by country, (%).

Admission year			2007	2008	2009	2010	2007–2010
In-hospital angiography		MINAP (UK)	22.9	27.9	35.5	38.8	31.8
		RIKS-HIA (Sweden)	60.4	62.7	66.8	69.8	64.8
		ACTION (US)	72.4	75.2	76.6	78.3	76.0
In-hospital PCI		MINAP (UK)	16.5	20.0	23.4	26.1	21.8
		RIKS-HIA (Sweden)	39.0	40.2	42.7	45.9	41.9
		ACTION (US)	41.8	43.3	44.2	45.5	43.9
Betablocker at discharge		MINAP (UK)	73.1	74.9	76.5	78.7	76.0
		RIKS-HIA (Sweden)	88.8	88.6	88.2	89.0	88.6
		ACTION (US)	90.1	89.4	89.1	89.1	89.4
Antiplatelet at discharge	Any	MINAP (UK)	94.2	94.0	94.7	95.4	94.6
		RIKS-HIA (Sweden)	93.6	94.4	95.0	94.9	94.5
		ACTION (US)	95.0	95.1	95.6	95.7	95.4
	Dual	MINAP (UK)	74.9	74.6	76.4	78.6	76.2
		RIKS-HIA (Sweden)	63.2	66.9	70.6	72.4	68.1
		ACTION (US)	64.7	65.1	66.9	68.0	66.4
Statin at discharge		MINAP (UK)	91.2	91.0	91.5	92.1	91.5
		RIKS-HIA (Sweden)	79.7	81.4	82.7	83.6	81.1
		ACTION (US)	82.0	83.7	85.4	85.9	84.5
ACEI or ARB at discharge		MINAP (UK)	77.1	77.5	79	80.6	78.7
		RIKS-HIA (Sweden)	65.5	68.3	69.9	72.1	68.8
		ACTION (US)	65.9	64.5	64.3	63.9	64.5

N = 137,009 for MINAP (UK), 45,069 for RIKS-HIA (Sweden), and 147,438 for ACTION (US). Abbreviations same as in Tables 1 and 2.

**Table 4:** Treatment of NSTEMI patients, stratified by whether they received an in-hospital PCI, by country, (%).

Admission year			Total	PCI performed	No PCI performed
Betablocker at discharge		MINAP (UK)	76.0	85.7	72.8
		RIKS-HIA (Sweden)	88.6	91.6	86.4
		ACTION (US)	89.4	92.0	86.9
Antiplatelet at discharge	Any	MINAP (UK)	94.6	99.2	93.1
		RIKS-HIA (Sweden)	94.5	99.5	90.7
		ACTION (US)	95.4	99.5	91.7
	Dual	MINAP (UK)	76.2	93.2	70.6
		RIKS-HIA (Sweden)	68.1	93.7	48.8
		ACTION (US)	66.4	94.4	40.7
Statin at discharge		MINAP (UK)	91.5	97.2	89.5
		RIKS-HIA (Sweden)	81.1	94.9	71.9
		ACTION (US)	84.5	91.1	78.5
ACEI or ARB at discharge		MINAP (UK)	78.7	87.7	75.7
		RIKS-HIA (Sweden)	68.8	75.2	64.0
		ACTION (US)	64.5	70.4	59.1

N = 137,009 for MINAP (UK), 45,069 for RIKS-HIA (Sweden), and 147,438 for ACTION (US). Abbreviations same as in Tables 1 and 2.
